# Investigating the electrical crosstalk effect between pixels in high-resolution organic light-emitting diode microdisplays

**DOI:** 10.1038/s41598-023-41033-4

**Published:** 2023-08-28

**Authors:** Haneul Kang, Yeonsu Hwang, Chan-mo Kang, Joo Yeon Kim, Chul Woong Joo, Jin-Wook Shin, Soobin Sim, Hyunsu Cho, Dae Hyun Ahn, Nam Sung Cho, Hyoc Min Youn, Young Jae An, Jin Sun Kim, Chun-Won Byun, Hyunkoo Lee

**Affiliations:** 1 Department of Electrical Engineering and Institute of Advanced Materials and Systems, Sookmyung Women’s University, Seoul, 04310 Republic of Korea; 2 Reality Display Research Section, Electronics and Telecommunications Research Institute (ETRI), Daejeon, 34129 Republic of Korea; 3 DONGJIN SEMICHEM CO., LTD, Hwaseong, 18635 Republic of Korea

**Keywords:** Electrical and electronic engineering, Organic LEDs, Electronic devices

## Abstract

Organic light-emitting diode (OLED) microdisplays have received great attention owing to their excellent performance for augmented reality/virtual reality devices applications. However, high pixel density of OLED microdisplay causes electrical crosstalk, resulting in color distortion. This study investigated the current crosstalk ratio and changes in the color gamut caused by electrical crosstalk between sub-pixels in high-resolution full-color OLED microdisplays. A pixel structure of 3147 pixels per inch (PPI) with four sub-pixels and a single-stack white OLED with red, green, and blue color filters were used for the electrical crosstalk simulation. The results showed that the sheet resistance of the top and bottom electrodes of OLEDs rarely affected the electrical crosstalk. However, the current crosstalk ratio increased dramatically and the color gamut decreased as the sheet resistance of the common organic layer decreased. Furthermore, the color gamut of the OLED microdisplay decreased as the pixel density of the panel increased from 200 to 5000 PPI. Additionally, we fabricated a sub-pixel circuit to measure the electrical crosstalk current using a 3147 PPI scale multi-finger-type pixel structure and compared it with the simulation result.

## Introduction

Organic light-emitting diodes (OLEDs) are used in various electronic devices and vehicles owing to their fast response time, high contrast ratio, wide color gamut, as well as ultrathin and flexible form factors^1–5^. OLED displays are available in different sizes and can be applied to various devices, from mobile phones to TVs^6,7^. In recent years, OLED microdisplays with a diagonal size of 2 in or less have been manufactured and applied to augmented reality (AR)/virtual reality (VR) devices^8^. The pixels in OLED microdisplays are magnified by optical systems in AR/VR devices^9,10^. Therefore, OLED microdisplays must have high pixel density. Most OLED microdisplays use silicon backplanes with complementary metal–oxide–semiconductor (CMOS)-based circuits for high resolution^8^. For instance, BOE in China reported a 5644 pixel per inch (PPI) OLED microdisplay for AR glass^11^. Sony also reported a small pixel pitch of 6.3 μm^12^.

**Figure 1:**
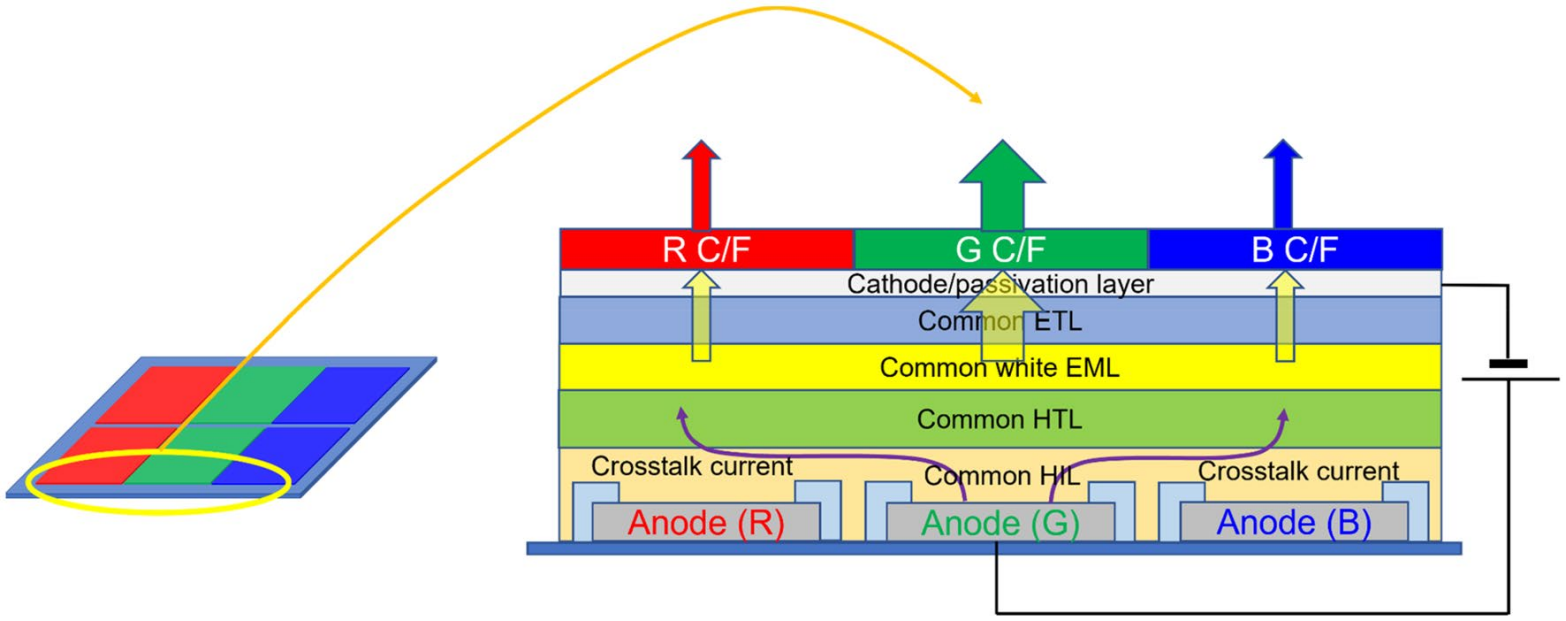
Schematic of crosstalk current in the full-color OLED microdisplay with R, G, and B color filters (C/Fs).

However, as the size of the pixel pitch decreases, the distance between pixels also decreases, resulting in problems^13,14^ that do not occur in conventional OLED displays. Because the distance between sub-pixels in the normal OLED display is large, approximately tens of micrometers, adjacent sub-pixels are not affected by lateral leakage current when a sub-pixel is electrically driven owing to the very high sheet resistance of organic materials. However, the distance between sub-pixels in the OLED microdisplay is approximately hundreds of nanometers^15,16^. Consequently, the driving voltage in the green (G) sub-pixel can cause a crosstalk current in the red (R) and blue (B) sub-pixels, called electrical crosstalk, as shown in Fig. 1^17,18^.

Although Liu et al. reported that lateral hole diffusion current improves the efficiency and operation stability of OLEDs^19^, most electrical crosstalk caused by lateral leakage current typically distorts pixel colors and decreases the color gamut of OLED microdisplay panels. White OLEDs with tandem structures are used because of their high luminance, efficiency, and lifetime in OLED microdisplays^20–22^. The tandem structure requires charge generation layers (CGLs)^23–25^, which have higher conductivity than normal organic materials, resulting in an electrical crosstalk^26^. Electrical crosstalk is a critical issue in the OLED microdisplay field and should be overcome to achieve a wide color gamut. However, experimental crosstalk current measurement and analysis are very difficult considering the size of the sub-pixel is very small, and practical OLED microdisplay panels are required.

This study investigates the effect of electrical crosstalk using a practical OLED microdisplay panel pixel structure. We conducted an electrical simulation and calculated the color gamut depending on the pixel parameters and structures, including the arrangement of color filters (C/Fs), the sheet resistance of the common organic layer, bottom and top electrodes, and the pixel densities. Additionally, we developed a lateral leakage current measurement circuit and measured the electrical crosstalk current on a practical OLED microdisplay pixel scale.

## Results and discussion

### Single stack white OLED for OLED microdisplay

**Figure 2:**
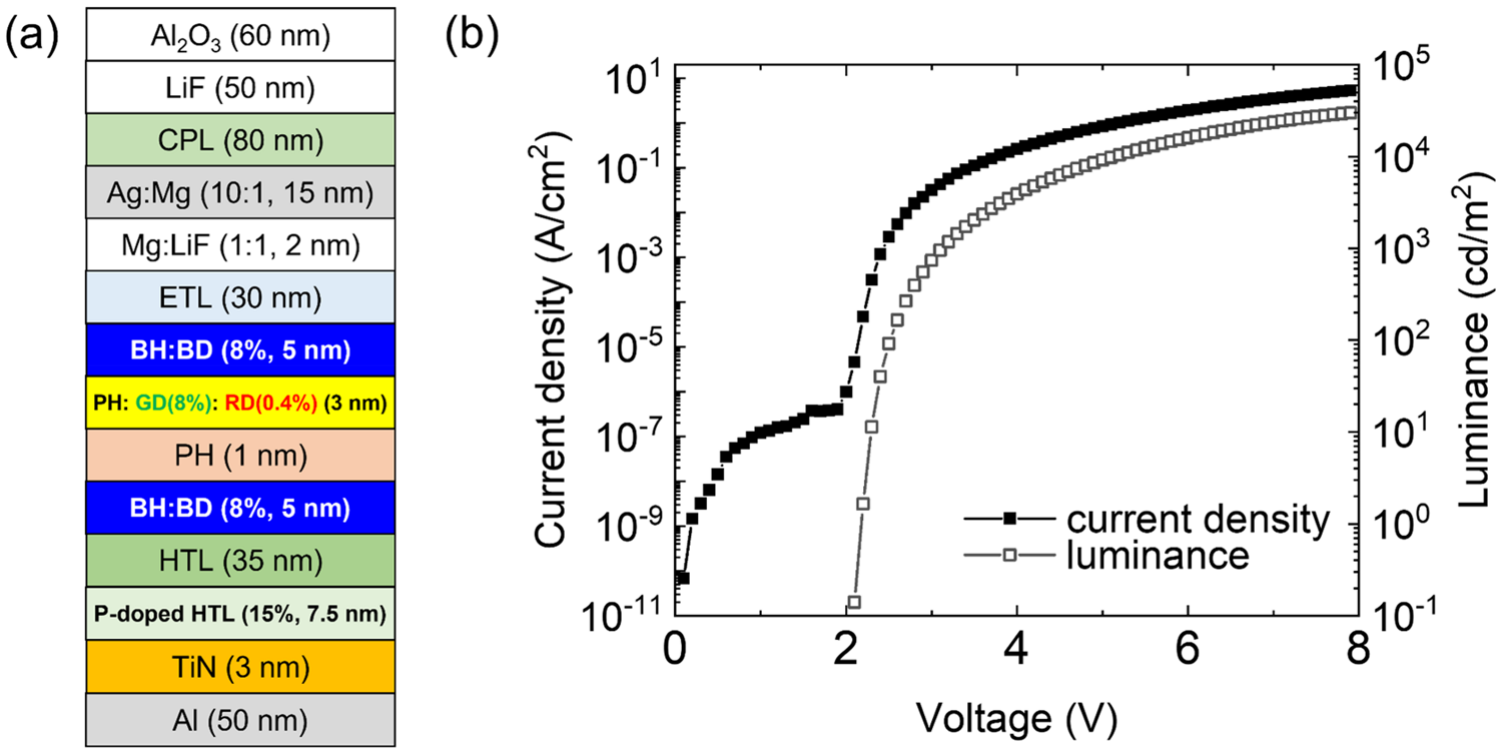
(**a**) Schematic device structure of white OLED for simulation (BH: blue host, BD: fluorescent blue dopant, GD: phosphorescent green dopant, RD: phosphorescent red dopant) and (**b**) *J–V–L* characteristics of the device.

A single-stack white OLED was fabricated for the electrical crosstalk simulation. Figure 2a shows a detailed structure of the white OLED: Si/Al (50 nm)/TiN (3 nm)/P-doped hole transport layer (HTL) (7.5 nm) as a hole injection layer (HIL)/HTL (35 nm)/blue emitting layer (EML) (5 nm)/phosphorescent host (PH) (1 nm)/phosphorescent green and red dopants co-doped EML (3 nm)/blue EML (5 nm)/electron transport layer (ETL) (30 nm)/Mg:LiF (1:1, 2 nm) as an electron injection layer (EIL)/Ag:Mg (10:1, 15 nm) as a semi-transparent cathode/CPL (80 nm) as a capping layer/LiF (50 nm) as a passivation layer/Al_2_O_3_ (60 nm) as a thin-film encapsulation layer. NDP-9 was used as a p-type dopant^24^. The energy levels of most materials and absorption and photoluminescence (PL) spectra of RD and BD were reported in our previously published paper^27^. Additionally, absorption and PL spectra of GD were provided in Fig. S1 in the Supplementary information. The material used for CPL is the same as that used for HTL. Figure 2b shows the current density–voltage–luminance (*J–V–L*) characteristics of the fabricated white OLED.

### Simulation process

Commercial software LAOSS (Fluxim) which used 2 + 1D finite element model based on the conductivity of the common layer^17,18^ and *J–V–L* characteristics of the fabricated white OLED were used for the electrical crosstalk calculation.

**Figure 3:**
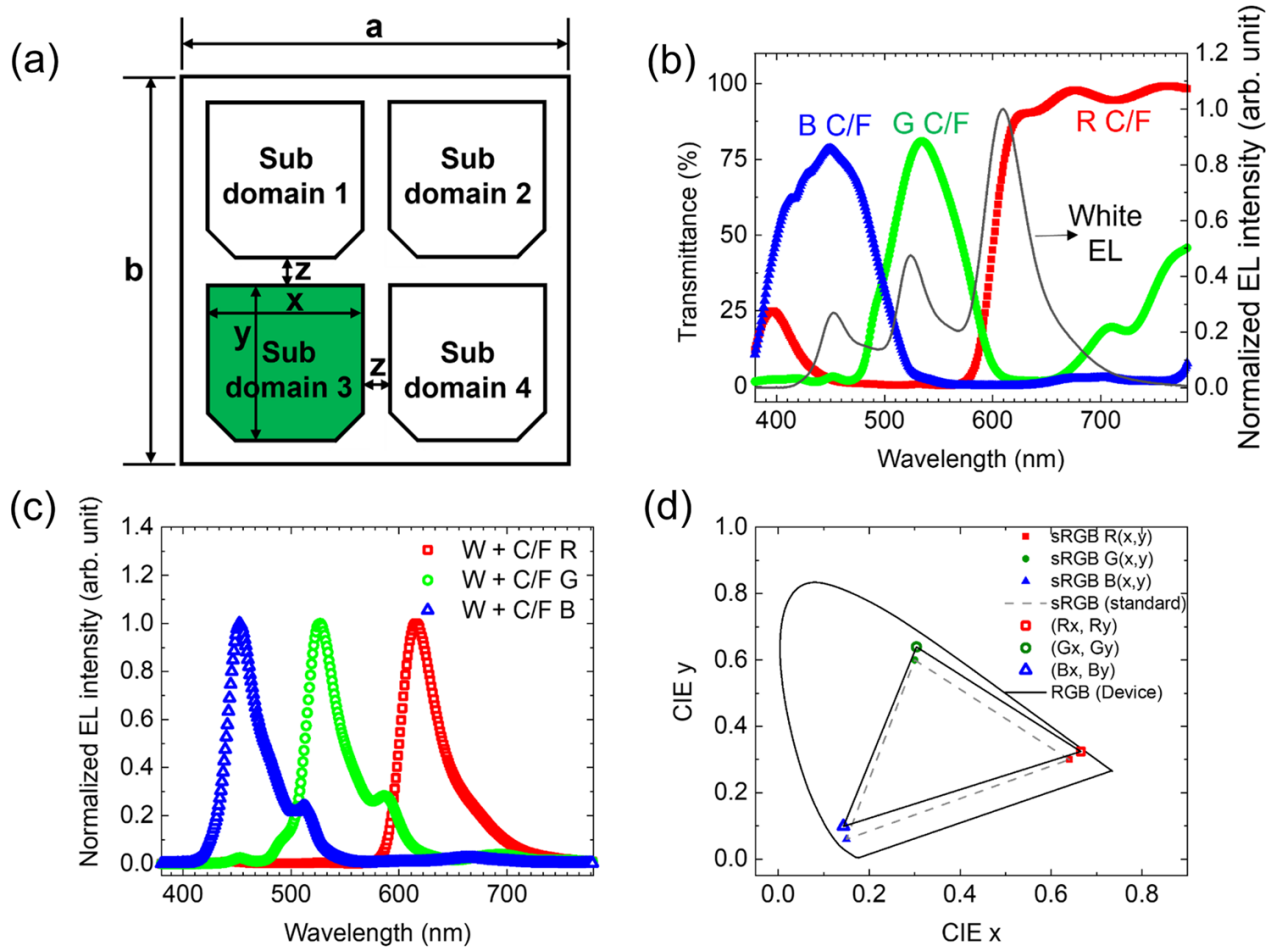
(**a**) Schematic pixel structure (a: 8.1 μm, b: 8.1 μm, x: 3.4 μm, y: 3.4 μm, z: 0.65 μm) for electrical crosstalk simulation, (**b**) transmittance of red, green, blue C/Fs and normalized EL spectrum of white OLED at 3.5 V, (**c**) red, green, and blue spectra of the white OLED through C/Fs, and (**d**) Commission Internationale de l’Eclairage (CIE) 1931 color coordinates of sRGB standard and the white OLED through C/Fs. The gray dashed line refers to the sRGB color space.

A pixel structure was designed based on a practical OLED microdisplay panel that was 0.7 in diagonally with 1920 × 1080 resolution, as shown in Fig. 3a. The pixel comprised four sub-pixels, and the pixel density was approximately 3147 PPI. When sub domain 3 sub-pixel was electrically driven, the electrical crosstalk currents of sub domai 1, 2, and 4 were calculated. The current crosstalk ratio was calculated as:

$$ \begin{aligned} & Current\,crosstalk\,ratio \; \left( \% \right) \\ & \quad = \frac{device\,current\,of\,sub\,domain\,for\,observing\,electrical\,crosstalk }{{device\,current\,of\,driving\,sub\,domain}} \times 100. \\ \end{aligned} $$

The luminance and white electroluminescence (EL) spectra of each sub-pixel were calculated using the current of each sub-pixel. Depending on the upper color filter arrangement, the white EL spectrum of each sub-pixel was multiplied by the transmittance of the color filter, as shown in Fig. 3b,c, to calculate the final EL spectra of each sub-pixel. By superimposing the calculated EL spectrum of each sub-pixel, we obtained the final EL spectrum and color coordinates of a pixel. By changing the driving sub domain, we calculated the color gamut as:

$$ \begin{aligned} & Color\,gamut \; \left( {sRGB} \right) \; (\% ) \\ & \quad = ABS\left( {\frac{{\left( {R_{x} - G_{x} } \right) \times B_{y} + \left( {G_{x} - B_{x} } \right) \times R_{y} + \left( {B_{x} - R_{x} } \right) \times G_{y} }}{2}} \right) \times 100/0.1121, \\ \end{aligned} $$

where (R_x_, R_y_), (G_x_, G_y_), and (B_x_, B_y_) are the color coordinates of the electrically driven red, green, and blue pixels, respectively, and 0.1121 is the area of the gray dashed triangle that comprises red, green, and blue color coordinates of sRGB as shown in Fig. 3d. We assumed that the sheet resistances of the bottom electrode, common organic layer, and top electrode were 2, 120 × 10^9^, and 20 Ω/□, respectively^18^.

### Color filter arrangement

**Figure 4:**
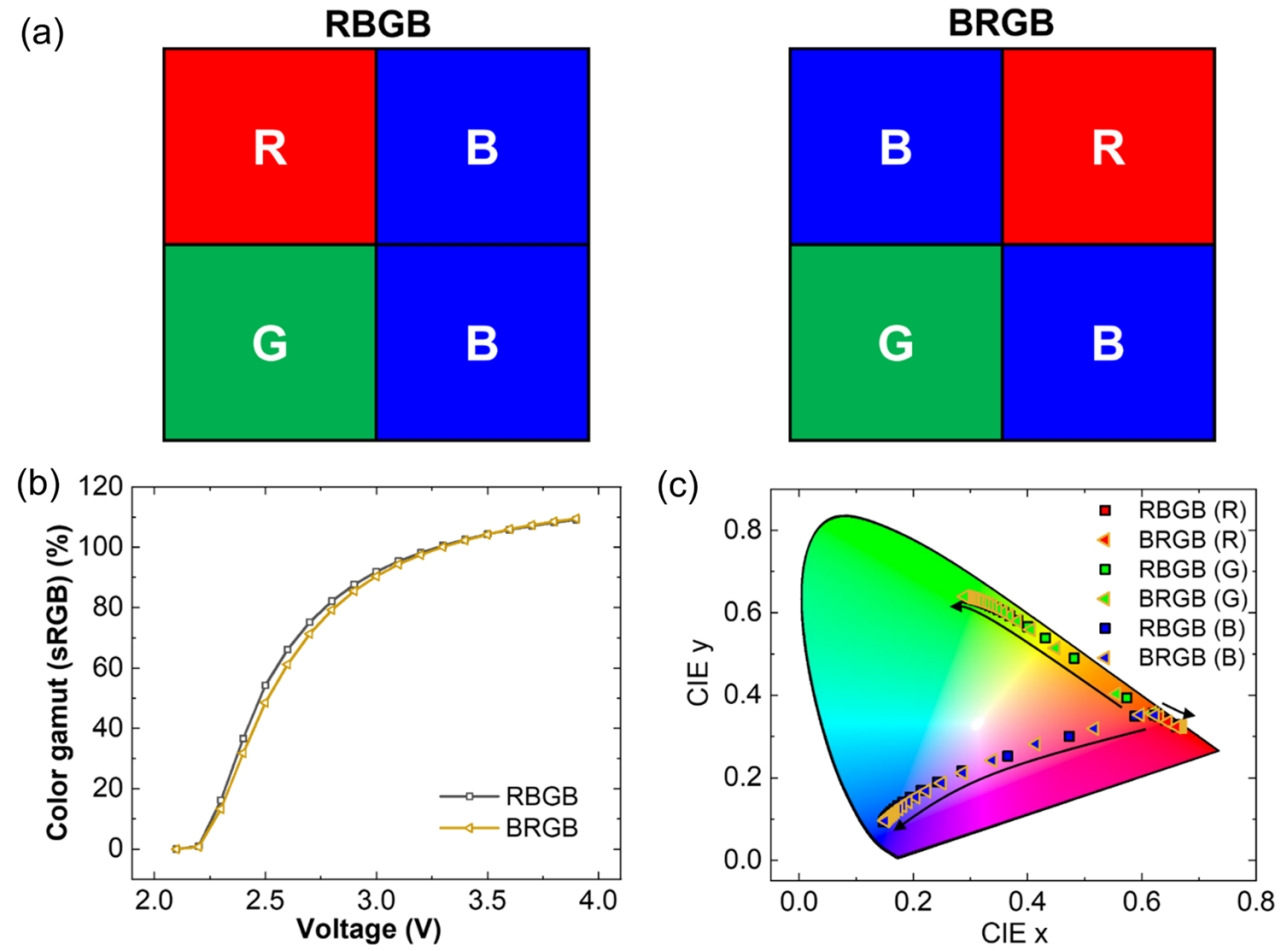
(**a**) Two different color filter arrangements, (**b**) calculated color gamut depending on the color filter arrangements with different driving voltages, and (**c**) calculated red, green, and blue CIE color coordinates as a function of driving voltage from 2.1 to 3.9 V (arrow direction: voltage increase).

Considering the pixel comprises four sub-pixels, two sub-pixels should have the same color in one pixel. Because fluorescent blue emission commonly has low efficiency and lifetime compared to phosphorescent green or red emission in OLEDs, we selected two blue sub-pixels, and one green and red sub-pixel each. Depending on the color filter arrangement, 12 different sub-pixel combinations were available. Among these, we selected two different color filter arrangements, as shown in Fig. 4a, since a common white OLED was used in the bottom layer. When voltage is applied to the red and green sub-pixels in the simulation, an almost identical current crosstalk ratio is observed at the same voltage, regardless of the color filter arrangement as shown in Fig. S2a,b in the Supplementary information. However, when voltage is applied to the blue sub-pixels, different current crosstalk ratios are exhibited depending on the color filter arrangement as shown in Fig. S2c in the Supplementary information. For example, when 2.5 V is applied to the blue sub-pixels, RBGB color filter arrangement showed higher lateral leakage current compared with BRGB color filter arrangement as shown in Fig. S2h,i in the Supplementary information. We calculated the color gamut based on sRGB using the calculated leakage current of each sub-pixel with different driving voltages as shown in Fig. 4b. The color gamut changed depending on the driving voltages owing to color coordinate changes of white light emission passing through the red, green, and blue color filters from the fabricated white OLED, as shown in Fig. 4c. The color gamut of RBGB arrangements was slightly higher than that of BRGB arrangements from 2.5 to 3.0 V. For example, the color gamut of RBGB and BRGB arrangements were 75.22% and 71.35% at 2.7 V, respectively, due to higher current crosstalk ratio of BRGB color filter arrangement compared with that of RBGB color filter arrangement. This result indicates that the horizontal or vertical arrangement of blue sub-pixels is more advantageous than the diagonal arrangement of blue sub-pixels for protecting the color gamut decrease owing to electrical crosstalk current.

### Effect of sheet resistance of top electrode and the common organic layer

**Figure 5:**
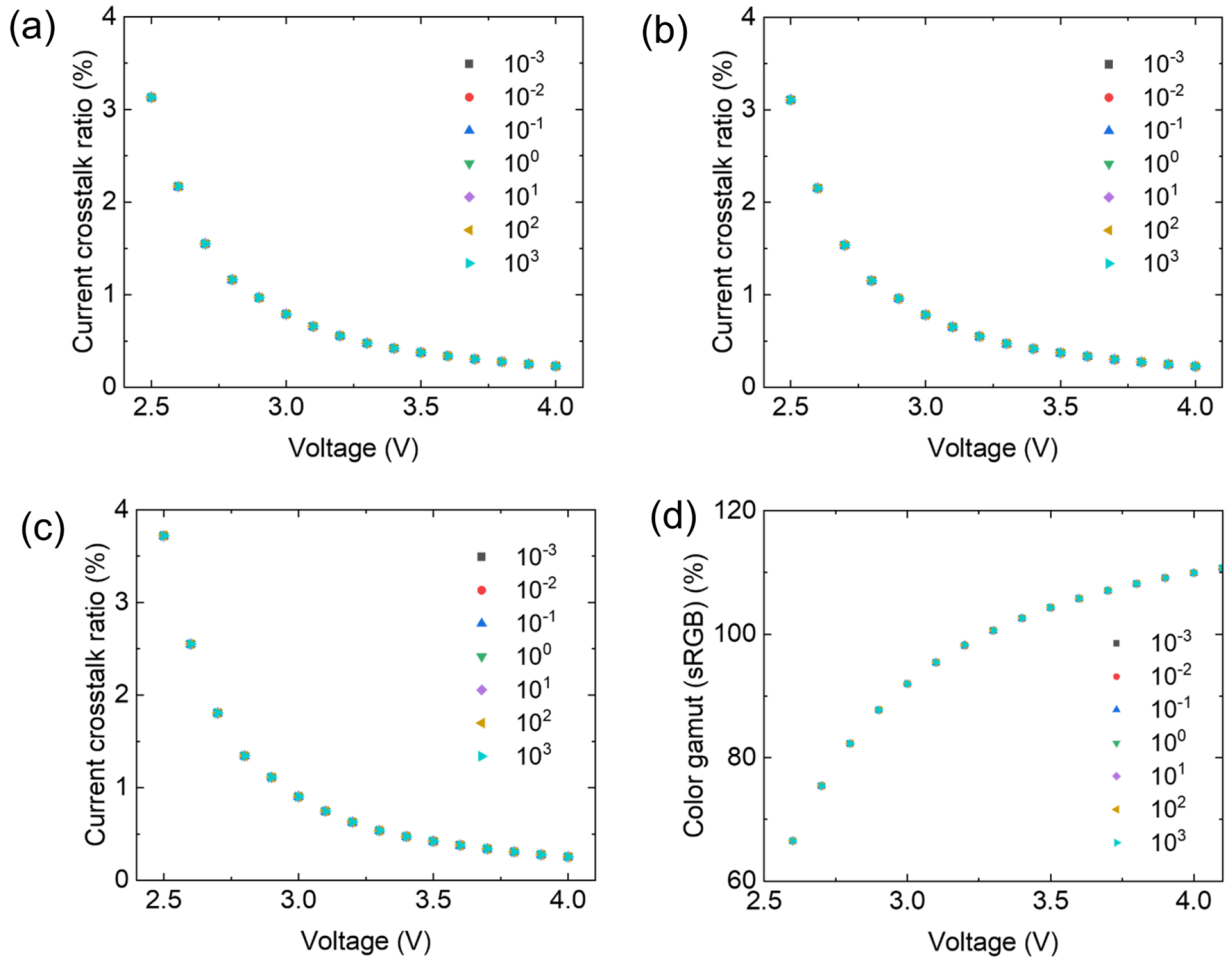
Current crosstalk ratio of (**a**) sub domain 1 on condition, (**b**) sub domain 3 on condition, (**c**) sub domain 2 and 4 on condition (**d**) and calculated color gamut with different sheet resistance (Ω/□) of top electrodes as a function of driving voltages.

OLED microdisplays typically use a Si wafer as a substrate, which is opaque. Therefore, the top-emission structure should be used for OLED microdisplays. When a top-emission structure is used for OLED, a semi-transparent thin metal layer is used as the top electrode. The thickness of the top electrode is very important. A thin metal layer increases the transmittance of the top electrode, which increases the sheet resistance, resulting in an increased driving voltage. Conversely, a thick metal layer decreases the sheet resistance of the top electrode, which decreases the transmittance of the top electrode, resulting in low luminance and efficiency. Therefore, the appropriate thickness of the top electrode is a significant factor for efficient top-emission white OLEDs. To investigate the electrical crosstalk effect depending on the sheet resistance of the top electrode, the sheet resistance of the top electrode was changed from 10^–3^ Ω/□ to 10^3^ Ω/□. The current crosstalk ratios of the pixels were nearly the same regardless of the sheet resistance of the top electrodes, as shown in Fig. 5a–c. Driving voltage is applied to the patterned bottom electrode, while the top electrode, functioning as a cathode, is in a ground state and located at the top of the OLED in this simulation. Therefore, it is anticipated that lateral leakage current will primarily occur at the interface between the bottom electrode and the common organic layer. Additionally, the sheet resistance of the top electrode is typically much lower compared to that of the common organic layers. Consequently, variations in the sheet resistance of the top electrode do not substantially impact the current crosstalk ratio. Therefore, the calculated color gamuts of the pixels were the same for different sheet resistances of the top electrodes, as shown in Fig. 5d. The effect of electrical crosstalk on the sheet resistance of the bottom electrode was negligible, as shown in Fig. S3 in the Supplementary information, indicating that the sheet resistance of the top and bottom electrodes does not affect electrical crosstalk in high-resolution displays.

**Figure 6:**
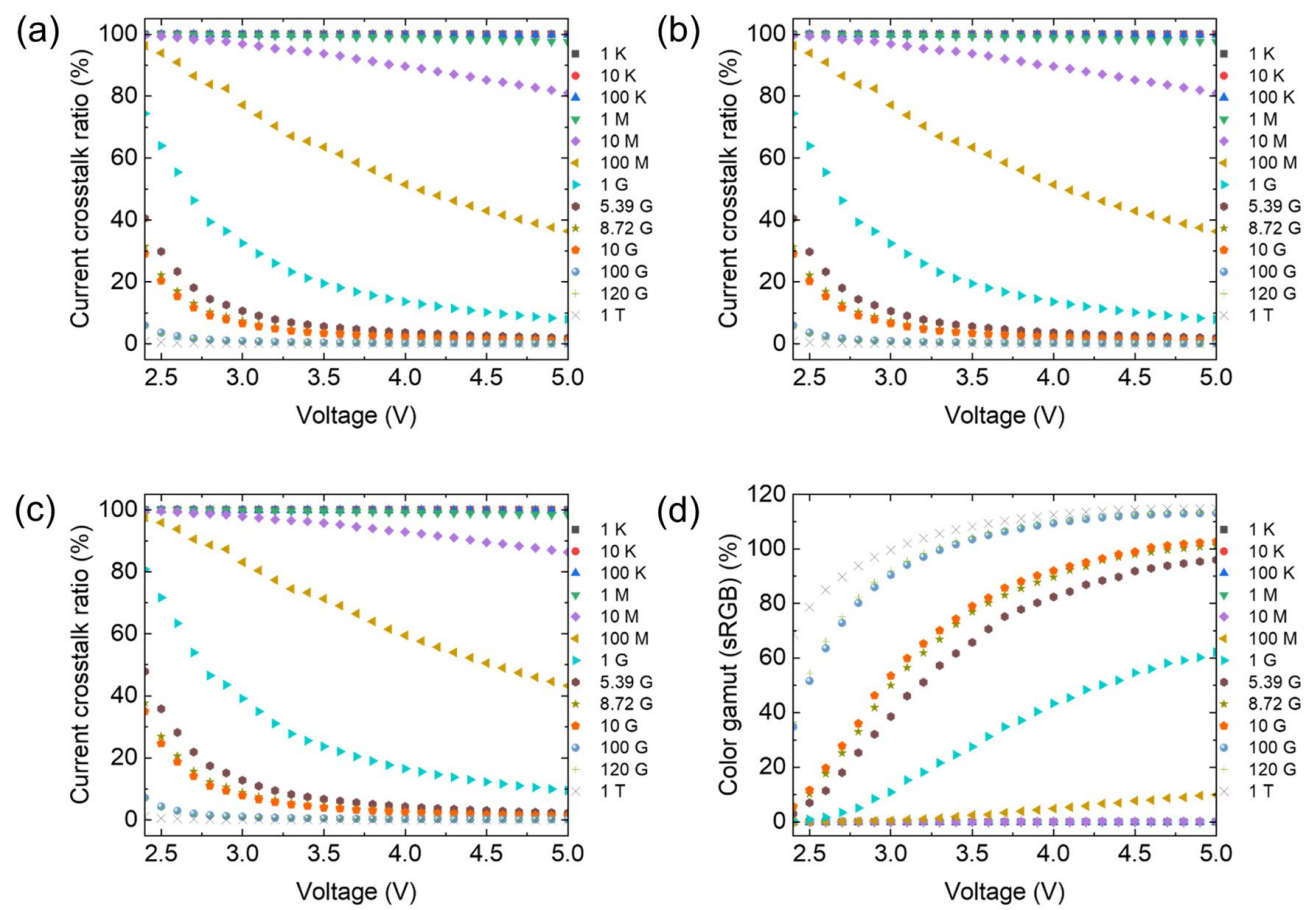
Current crosstalk ratio of (**a**) sub domain 1 on condition, (**b**) sub domain 3 on condition, (**c**) sub domain 2 and 4 on condition, and (**d**) calculated color gamut with different sheet resistance (Ω/□) of the common organic layer as a function of driving voltages.

An OLED comprises many organic layers between the electrodes and has very high resistance. However, in recent years, the conductivity of organic layers in OLED has been increased for low driving voltages by developing novel organic materials and using an n-type and p-type doping method^28–31^. Therefore, to investigate the effect of the resistance of organic layers, while considering the electrical crosstalk between pixels, we calculated the current crosstalk ratios and color gamut by changing the sheet resistance of the common organic layer from 1 KΩ/□ to 1 TΩ/□, as shown in Fig. 6. The sheet resistance of the organic layer critically affects the electrical crosstalk between pixels. For example, the current crosstalk ratio of the pixel is approximately 100% when the sheet resistance is between 1 kΩ/□ to 1 MΩ/□; therefore, the color gamut is approximately 0%. Even a 1 GΩ/□ sheet resistance results in a very low color gamut of 43.35% at 4.0 V. Considering n-type and p-type doped organic layers are typically used as CGLs in a tandem OLED structure, the conductivity of CGL is typically higher than that of other organic layers. We measured the sheet resistance of the n-type doped ETL and p-type HTL/n-type ETL layers using a 4-point probe method, as shown in Fig. S4 in the Supplementary information. The sheet resistances of these layers were 5.39 GΩ/□ and 8.72 GΩ/□, respectively, which can cause electrical crosstalk in tandem OLEDs, thereby decreasing the color gamut. As the sheet resistance of the organic layer increased, the current crosstalk ratio decreased, and the color gamut increased, indicating that the conductivity of the common organic layer in an OLED is crucial for determining electrical crosstalk in high-resolution pixel structures.

### Effect of pixel density

**Figure 7:**
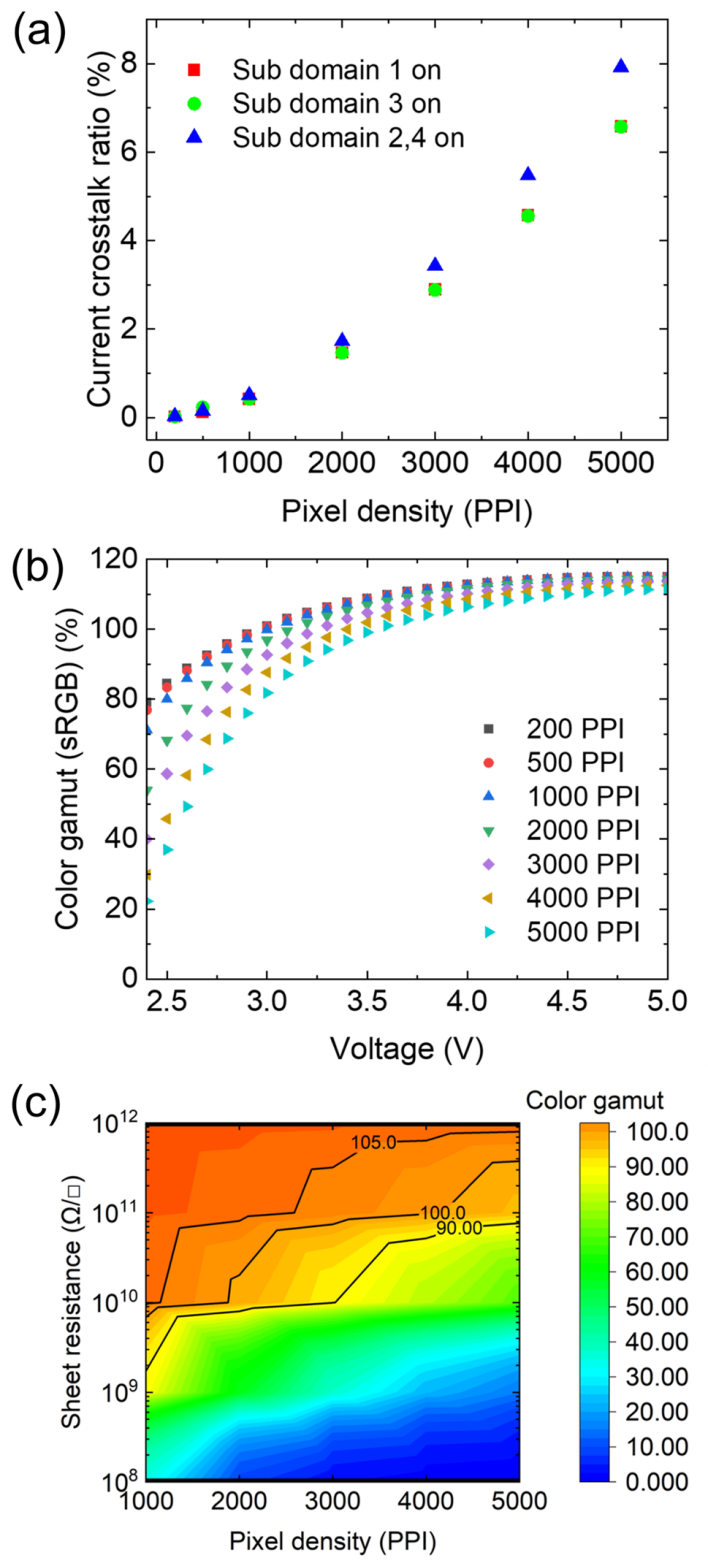
(**a**) Current crosstalk ratio with different pixel densities at 2.5 V, (**b**) driving voltage dependency, and (**c**) common organic layer sheet resistance dependency at 3.5 V of calculated color gamut with different pixel densities.

We fixed the pixel shape, panel resolution, and aperture ratio to 68.6%, which is the same as that of the practical 3147 PPI panel, and changed the pixel size and space for designing pixel structures with different pixel densities: 200 PPI, 500 PPI, 1000 PPI, 2000 PPI, 3000 PPI, 4000 PPI, and 5000 PPI. The spaces between the sub-pixels were 10.18 μm, 4.06 μm, 2.04 μm, 1.02 μm, 0.68 μm, 0.52 μm, and 0.41 μm, respectively. We calculated the current crosstalk ratios and color gamut based on the pixel densities. As the pixel density increased, the current crosstalk ratio increased, thereby decreasing the color gamut, as shown in Fig. 7a,b. Additionally, the sheet resistance of the common organic layer should be increased to reduce the decrease in color gamut owing to electrical crosstalk caused by the increased in pixel density, as shown in Fig. 7c. For example, approximately 7 × 10^9^ Ω/□ of the common organic layer sheet resistance is required for approximately 100% color gamut at a pixel density of 1000 PPI, whereas approximately 4 × 10^11^ Ω/□ of the common organic layer sheet resistance is required for approximately 100% color gamut at 5000 PPI pixel density. Therefore, the electrical crosstalk effect should be considered for a high color gamut when designing high-resolution OLED display pixels.

### Electrical crosstalk current measurement in practical OLED microdisplay pixel scale

**Figure 8:**
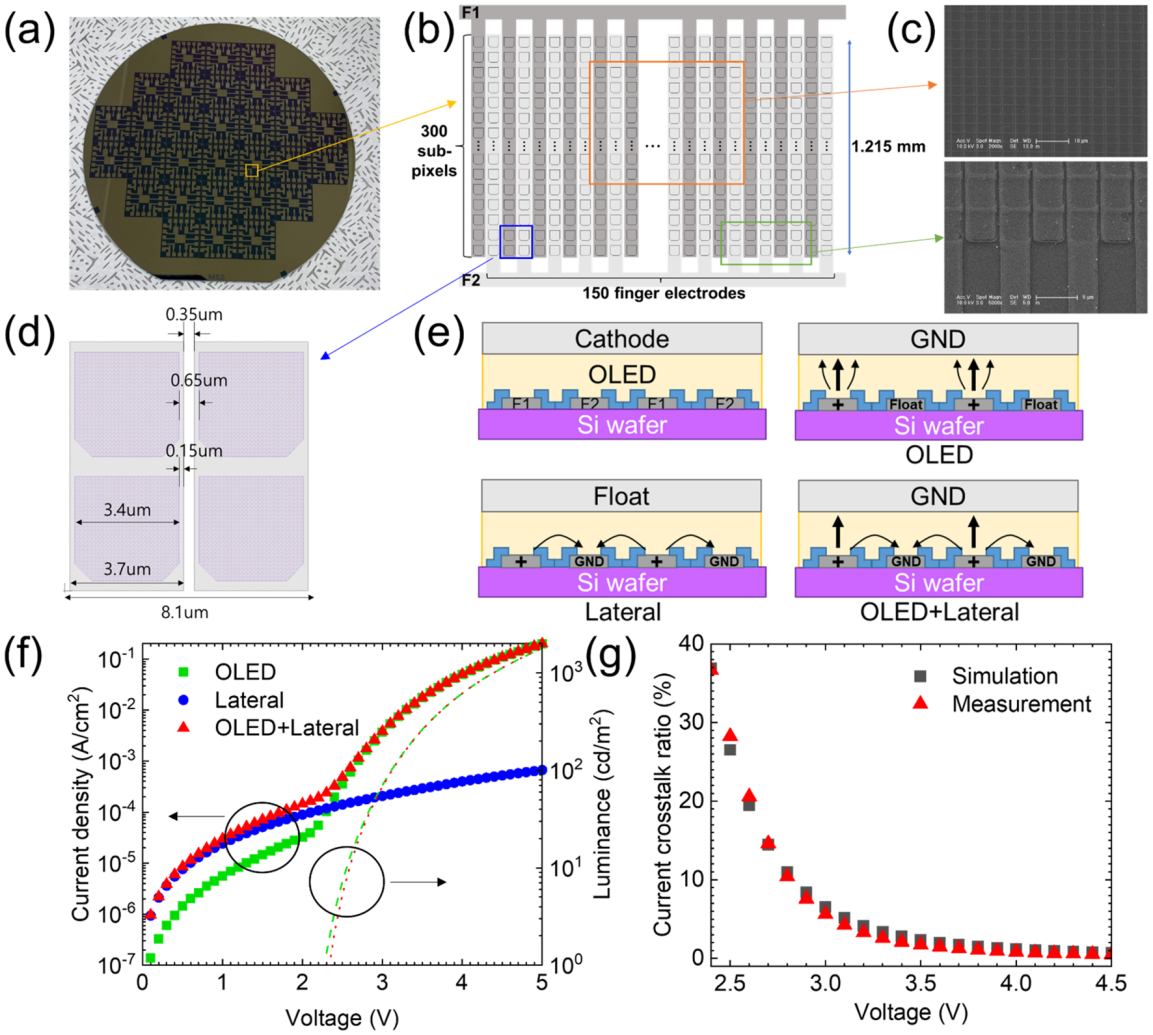
(**a**) Fabricated Si wafer with pixel circuit image for measuring the electrical crosstalk current, (**b**) schematic circuit structure with finger electrodes, (**c**) scanning electron microscope (SEM) image of finger electrodes with sub-pixels, (**d**) dimension of sub-pixels, (**e**) schematic cross-sectional structure of finger electrodes with different driving conditions, (**f**) *J–V–L* characteristics of single stack white OLED with different driving conditions, and (**g**) simulated and measured current crosstalk ratios depending on the driving voltages.

To measure the electrical crosstalk current and compare it with the simulation results, we fabricated a sub-pixel circuit on the Si wafer, as shown in Fig. 8a. The bottom electrode of the sub-pixel was Ti (2 nm)/Al (50 nm)/indium tin oxide (ITO) (5 nm). The white OLED structure was the same as that shown in Fig. 2a. The *J–V–L* characteristics of the device, as shown in Figure S5 in the Supplementary information, were used for the simulation. The bottom electrode was designed as a multi-finger-type structure to examine the lateral leakage between adjacent sub-pixels, as shown in Fig. 8b. A 30 nm thick SiO_2_ sub-pixel define layer with a metal opening area of approximately 3.4 µm × 3.4 µm was used, as shown in Fig. 8c,d.

Three driving conditions were used, as shown in Fig. 8e. In the “OLED” driving condition, the driving voltage applied to the F1 and F2 electrodes was in the floating state while the top cathode was in the ground state. In the “Lateral” driving condition, the driving voltage applied to the F1 and F2 electrodes was in the ground state and the top cathode was in the floating state. In “OLED + Lateral” driving condition, driving voltage was applied to F1 and F2 electrodes and top cathode were ground state. Figure 8f shows the *J–V–L* characteristics depending on the driving conditions. In the “Lateral” driving condition, OLED does not emit light because the current flows from the F1 electrode to the F2 electrode. The “OLED + Lateral” driving condition showed higher current density compared to that of the “OLED” driving condition in the low driving voltage region. The current density difference between the “OLED + Lateral” and “OLED” driving conditions was similar in terms of the “Lateral” driving condition, thereby indicating that a lateral leakage current exists between sub-pixels in high-resolution OLED microdisplays. To investigate the transport property of a lateral leakage current, hole-only devices (HODs) were fabricated with the following structures: ITO(150 nm)/P-doped HTL (15%, 160 nm)/Al(100 nm) and ITO(150 nm)/ 1,4,5,8,9,11-hexaazatriphenylene hexacarbonitrile (HAT-CN) (10 nm)/HTL (150 nm)/Al(100 nm) as shown in Fig. S6a in the Supplementary information. The HOD with P-doped HTL shows much higher current density compared with that of the HOD with pristine HTL as shown in Fig. S6b in the Supplementary information. For instance, the current density of the HOD with P-doped HTL is 1.14 A/cm^2^ which is approximately 419 times higher value compared with that of the HOD with pristine HTL. When calculating the conductivity of P-doped HTL using *J* = *σE* equation, the conductivity is approximately 6.2 × 10^–6^ S/cm. Therefore, the P-doped HTL, which serves as the HIL, contributes the most to the current conduction in the “Lateral” driving condition. The luminance of the “OLED” driving condition is slightly higher compared to that of the “OLED + Lateral” driving condition at the same voltage owing to the lateral leakage current of the “OLED + Lateral” driving condition. The calculated current crosstalk ratios using the measured lateral leakage currents with different driving voltages and the simulation results are shown in Fig. 8g. The current crosstalk ratio decreased as the driving voltage increased, and the simulation results matched well with the calculation results from the measurement data.

To reduce the lateral leakage current, a pixel structure with a gap spacer over a bank between adjacent pixels was reported^26^. In our sub-pixel structure, the thickness of the sub-pixel define layer was increased from 30 to 80 nm, as depicted in Fig. S7a–d in the Supplementary information. As the sub-pixel define layer thickness increases, the current density in the 'Lateral' driving condition decreases, as shown in Fig. S7e in the Supplementary Information, due to the reduced sheet resistance of the common organic layers. Consequently, controlling the resistance of the common organic materials and employing various pixel structures can be effective approaches to reducing lateral leakage current.

## Conclusion

We investigated the electrical crosstalk effect in high-resolution OLED microdisplay pixel arrays by calculating their current crosstalk ratios and color gamut. The horizontal or vertical arrangement of blue sub-pixels was found to be more advantageous than the diagonal arrangement of blue sub-pixels in protecting the decrease in color gamut by the electrical crosstalk current. The sheet resistance of the top and bottom electrodes did not affect the electrical crosstalk, whereas that of the common organic layer dramatically affected the electrical crosstalk in high-resolution OLED microdisplays. As the pixel density increased, the current crosstalk ratio increased and the color gamut decreased. Additionally, we fabricated and measured the lateral leakage current in a practical OLED microdisplay pixel scale using a multifinger-type circuit. The crosstalk current ratio from the measured current matched the simulation results well. Furthermore, increasing the thickness of the sub-pixel define layer reduced the lateral leakage current. Therefore, the sheet resistance of the common organic material and pixel structure are highly significant factors in determining the electrical crosstalk effect. We believe that these results will be helpful in designing and enhancing the performance of full-color high-resolution OLED microdisplays.

## Materials and methods

### Device fabrication process

CMOS-process-based Si substrates were used to fabricate single-stack white OLEDs. The substrate size was 2 cm × 2 cm and the active area was approximately 2 mm × 2 mm with a pixel pitch of 10.8 µm × 3.6 µm. The top metal of the Si substrate was Al/TiN. For the lateral leakage current measurement, 6 in Si substrates with thermally grown SiO_2_ (500 nm) were used. The active area of the OLED was 1.215 mm × 1.215 mm. For HODs, ITO patterned glass substrates were used. The substrates were sequentially rinsed with acetone, methanol, and deionized water for 15 min each, and dried in a vacuum oven at 80 °C. Subsequently, the organic and metal layers were deposited using a vacuum thermal evaporator to fabricate the OLEDs. A 60 nm thick Al_2_O_3_ layer was deposited onto the OLEDs using atomic layer deposition (ALD) to protect the OLEDs from moisture and oxygen. The ALD process temperature was maintained at 95 °C. Red, green, and blue color filters were supplied by DONGJIN SEMICHEM Co., Ltd.

### Device and film characterization

The *J–V* characteristics of the devices were measured in a dark room at room temperature using a source measurement unit (Keithley 238 and 2450). Luminance (*L*), *EL* spectra, CIE color coordinates were measured using a spectroradiometer (Konica Minolta CS-2000). The transmittance of the color filters was measured using a UV–Vis–NIR spectrophotometer (PerkinElmer LAMBDA 750). The absorption spectrum was measured using a UV/Vis spectrophotometer (UV-2550, Shimazu, Japan). The PL spectra were measured using an UV lamp (VL-6.LC, VILBER, France) with excitation wavelength of 254 nm with 6 W and a spectroradiometer (CS-2000, Konica Minolta, Japan). The field emission scanning electron microscopes (JSM-7600F, Jeol, Japan and Sirion400, FEI, U.S.A.) were used for SEM images.

### Supplementary Information

Supplementary Figures.

## Data Availability

The data that support the findings of this study are available from the corresponding author upon reasonable request.
