# Supplementary material for: Investigating the electrical crosstalk effect between pixels in high-resolution organic light-emitting diode microdisplays
Source: Sci Rep. 2023 Aug 28;13:14070. doi: 10.1038/s41598-023-41033-4 (PMC10462745; doi:10.1038/s41598-023-41033-4)
Supplement: Supplementary file 1 — Supplementary Figures. [file 41598_2023_41033_MOESM1_ESM.docx]

Supplementary Information

Investigating the electrical crosstalk effect between pixels in high-resolution organic light-emitting diode microdisplays

Haneul Kang^1,†^, Yeonsu Hwang^1,†^, Chan-mo Kang^2^, Joo Yeon Kim^2^, Chul Woong Joo^2^, Jin-Wook Shin^2^, Soobin Sim^1^, Hyunsu Cho^2^, Dae Hyun Ahn^2^, Nam Sung Cho^2^, Hyoc Min Youn^3^, Young Jae An^3^, Jin Sun Kim^3^, Chun-Won Byun^2^, and Hyunkoo Lee^1,^*

^1^ Department of Electrical Engineering and Institute of Advanced Materials and Systems, Sookmyung Women’s University, Seoul 04310, Republic of Korea

^2^Reality Display Research Section, Electronics and Telecommunications Research Institute (ETRI), Daejeon 34129, Republic of Korea

^3^DONGJIN SEMICHEM CO., LTD, Hwaseong 18635, Republic of Korea

^†^These authors contributed equally to this work.

*Corresponding author:

Hyunkoo Lee

E-mail: lhk108@sookmyung.ac.kr


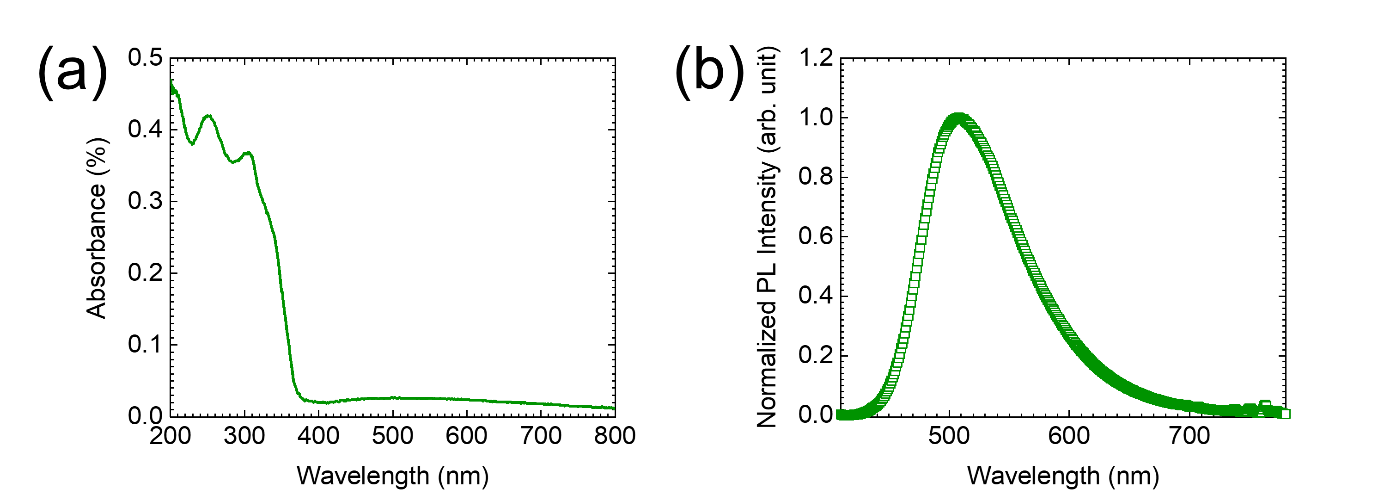


Figure S1. (a) Absorption and (b) photoluminescence (PL) spectra of the phosphorescent green dopant.


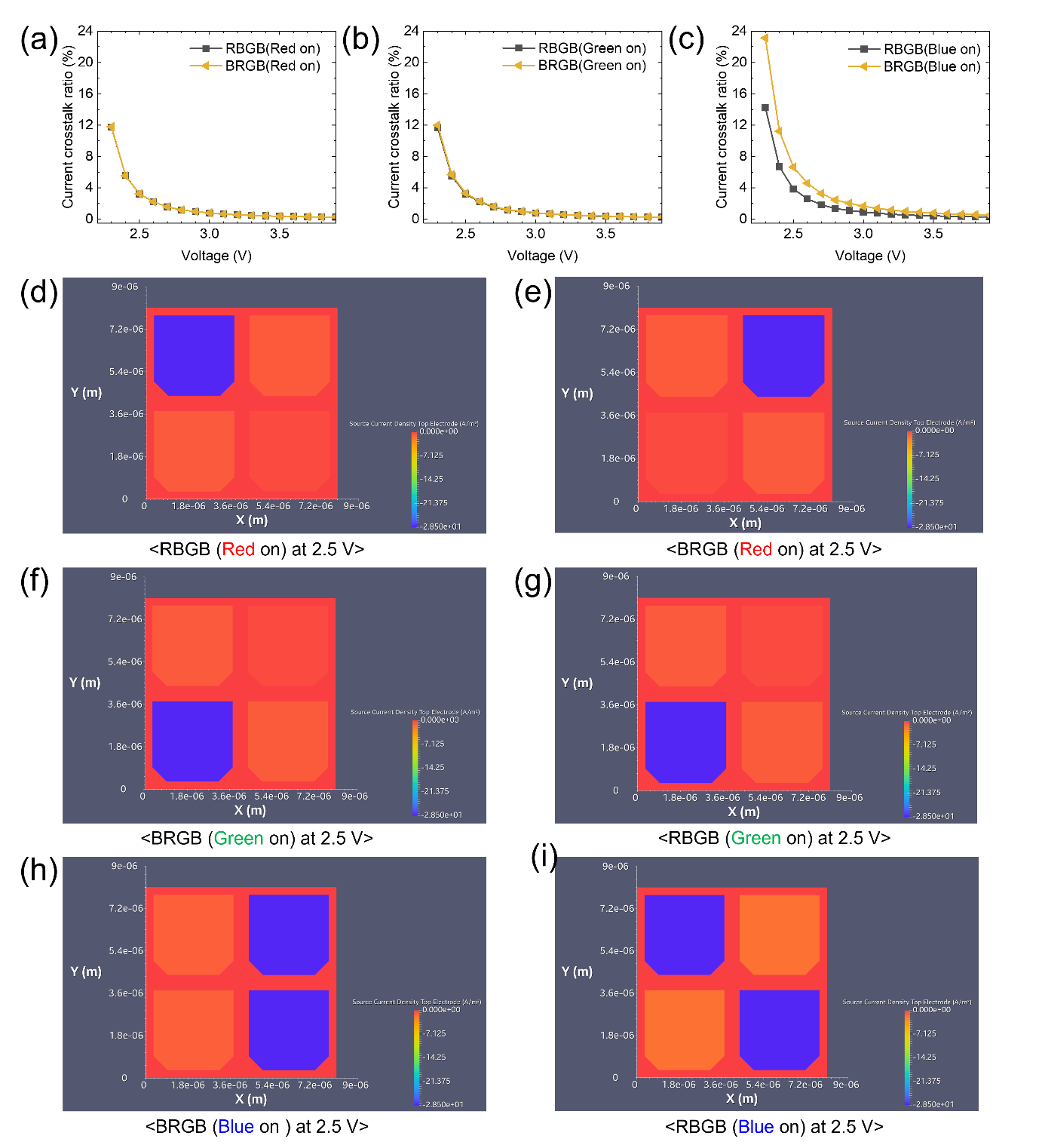


Figure S2. Current crosstalk ratio of (a) red sub-pixel on condition, (b) green sub-pixel on condition, (c) blue sub-pixels on condition with RBGB and BRGB color filter arrangements, current density through the OLED at 2.5 V of (d) red sub-pixel on (RBGB), (e) red sub-pixel on (BRGB), (f) green sub-pixel on (BRGB), (g) green sub-pixel on (RBGB), (h) blue sub-pixels on (BRGB), and (i) blue sub-pixels on (RBGB).


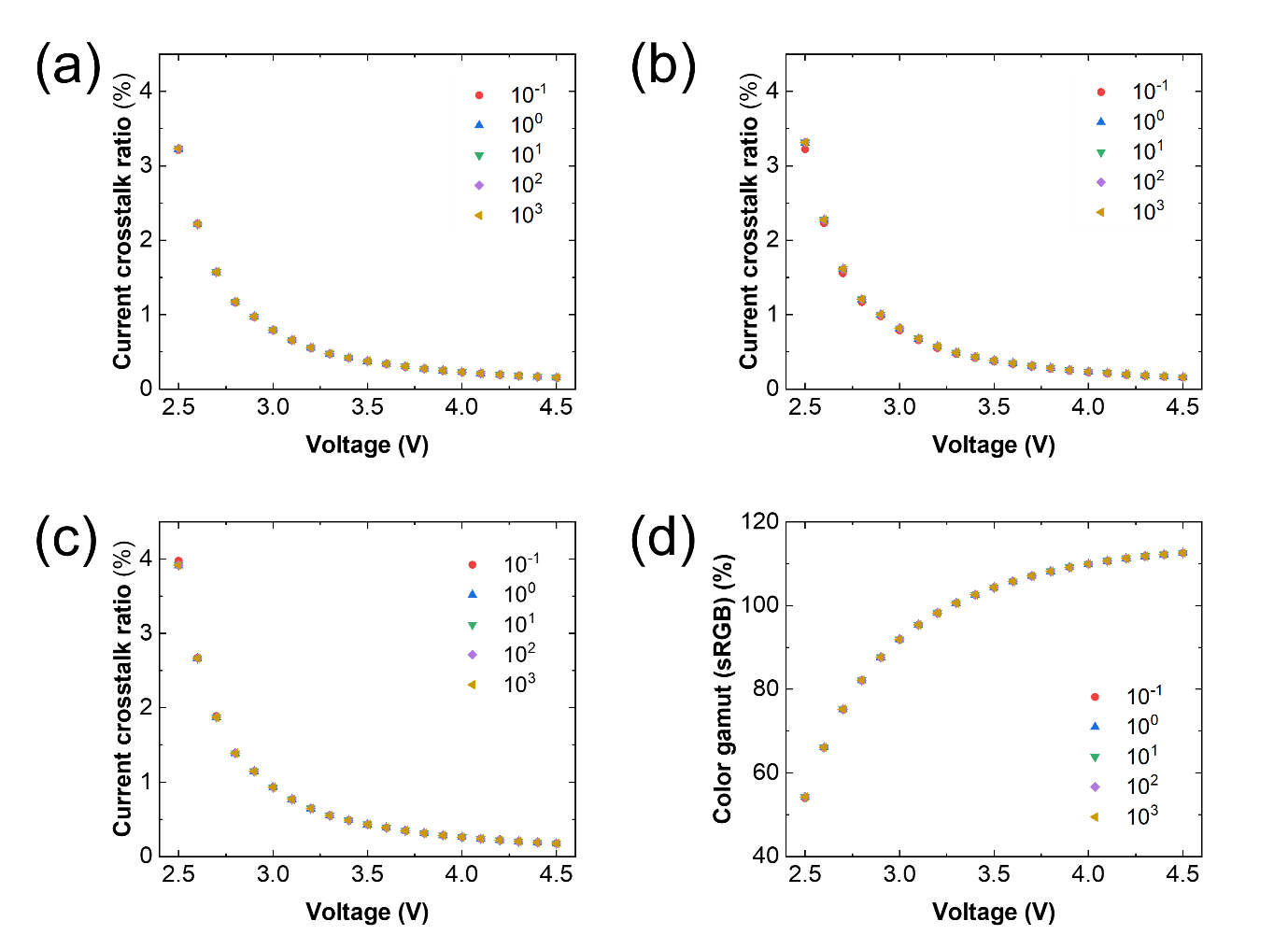


Figure S3. Current crosstalk ratio of (a) sub domain 1 on condition, (b) sub domain 3 on condition, (c) sub domain 2 and 4 on condition, and (d) calculated color gamut with different sheet resistance (Ω/□) of bottom electrodes as a function of driving voltages.


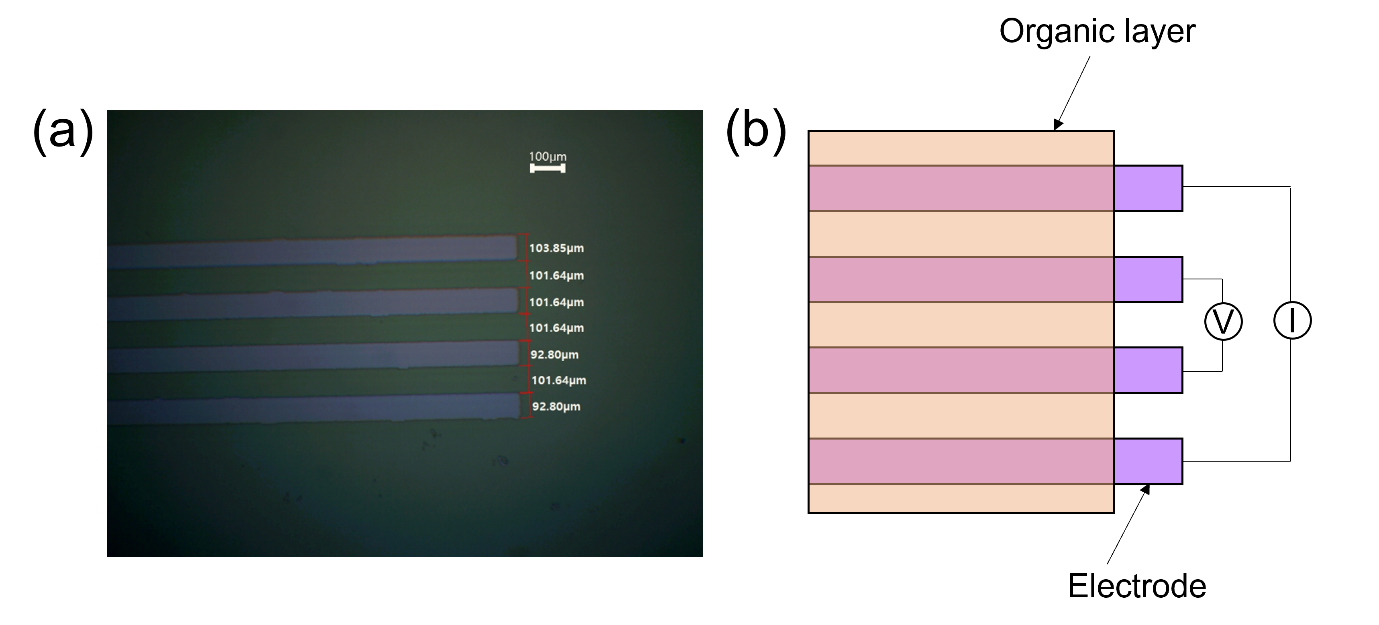


Figure S4. (a) ITO pattern and (b) 4-point probe circuit for measuring the sheet resistance of Li-doped ETL (100 nm) and p-type doped HTL (10 nm)/Li-doped ETL (100 nm).


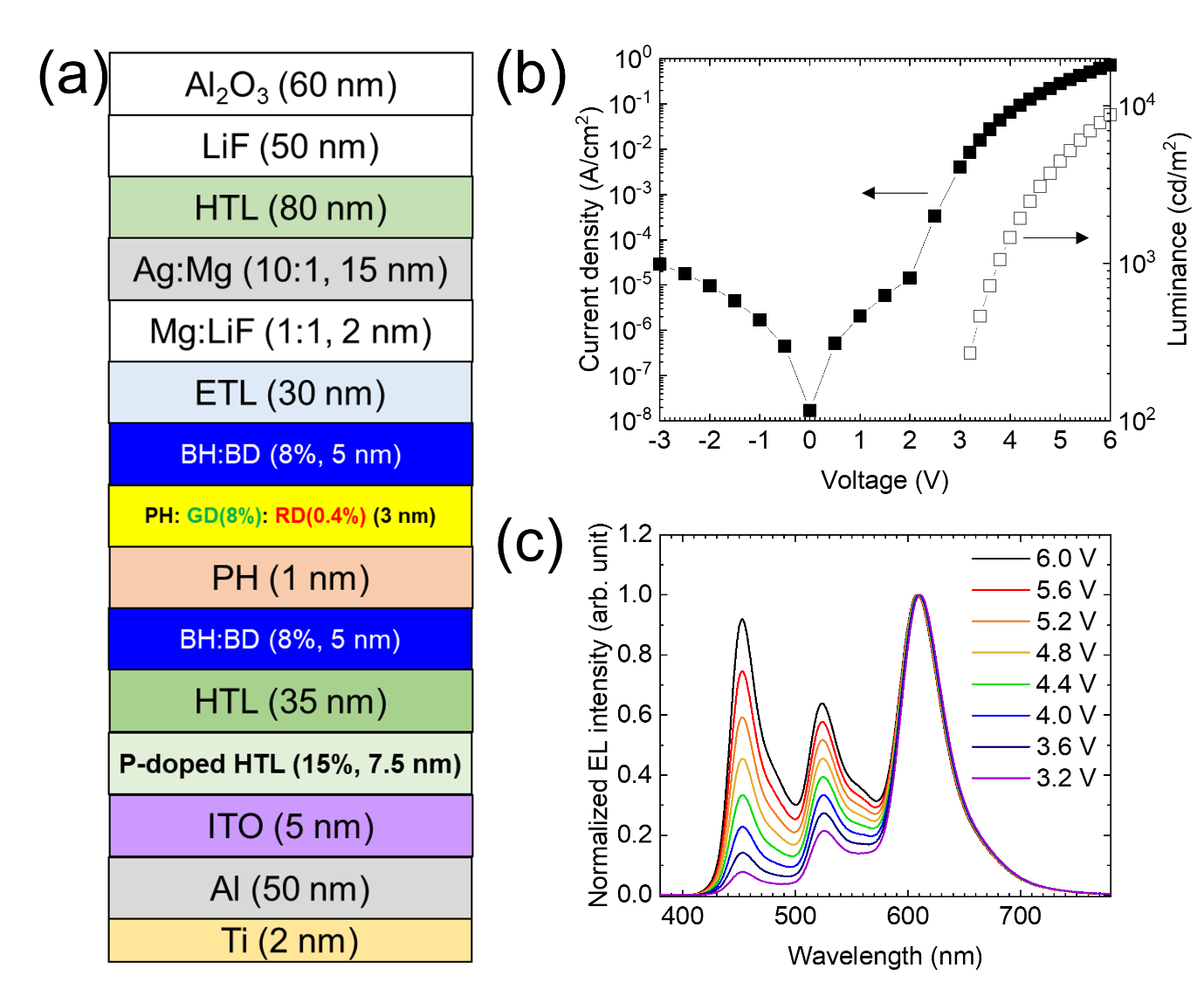


Figure S5. (a) Schematic device structure of a white OLED for electrical crosstalk measurement and simulation (b) current density–voltage–luminance (*J–V–L*) characteristics, and (c) EL spectra depending on driving voltages.


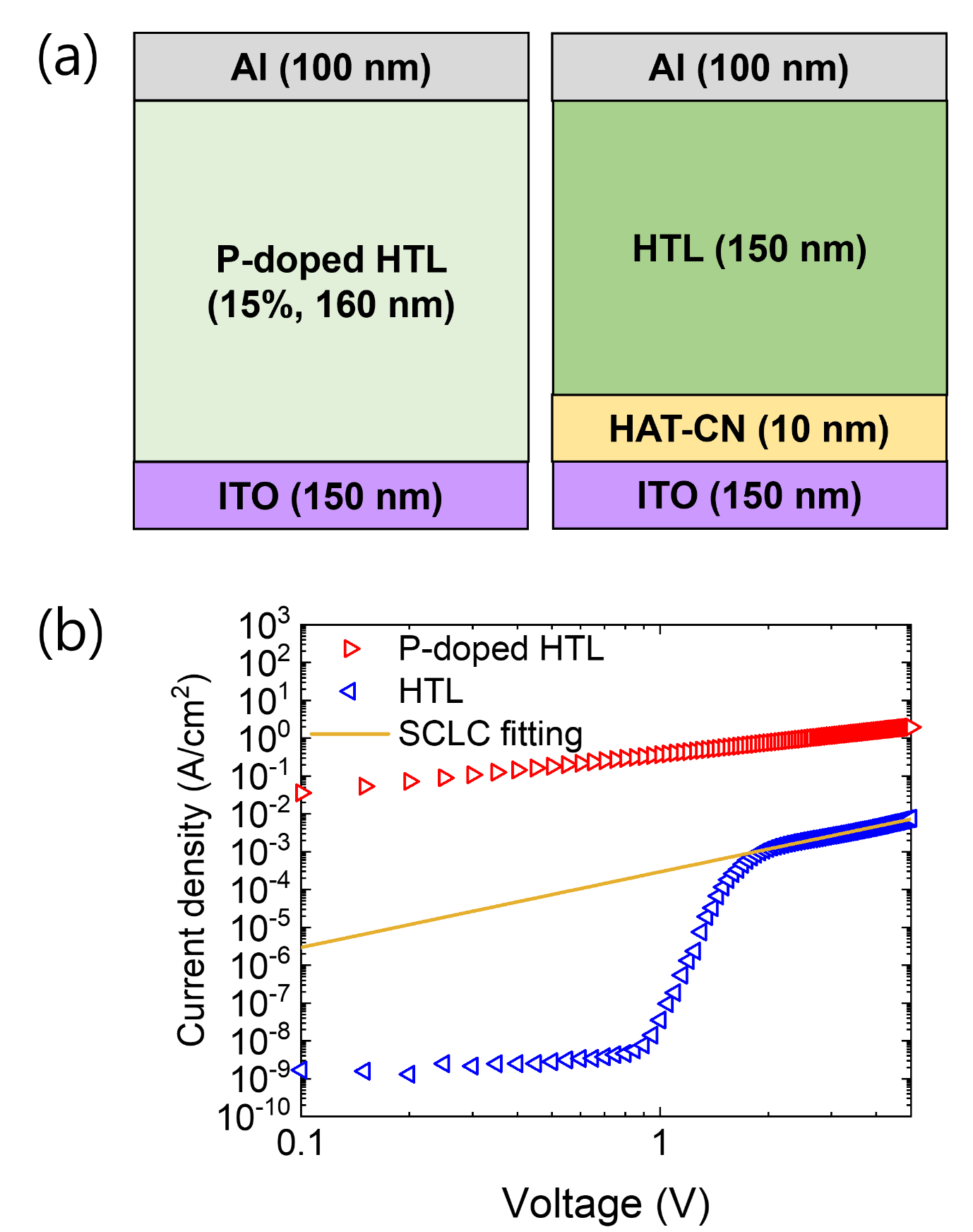


Figure S6. (a) Schematic structure of hole-only devices (HODs) and (b) current density–voltage (*J–V*) characteristics of HODs. The calculated hole mobility of HTL by SCLC fitting is approximately 3.43×10^-6^ cm^2^/V·sec at 250 kV/cm^S1^.


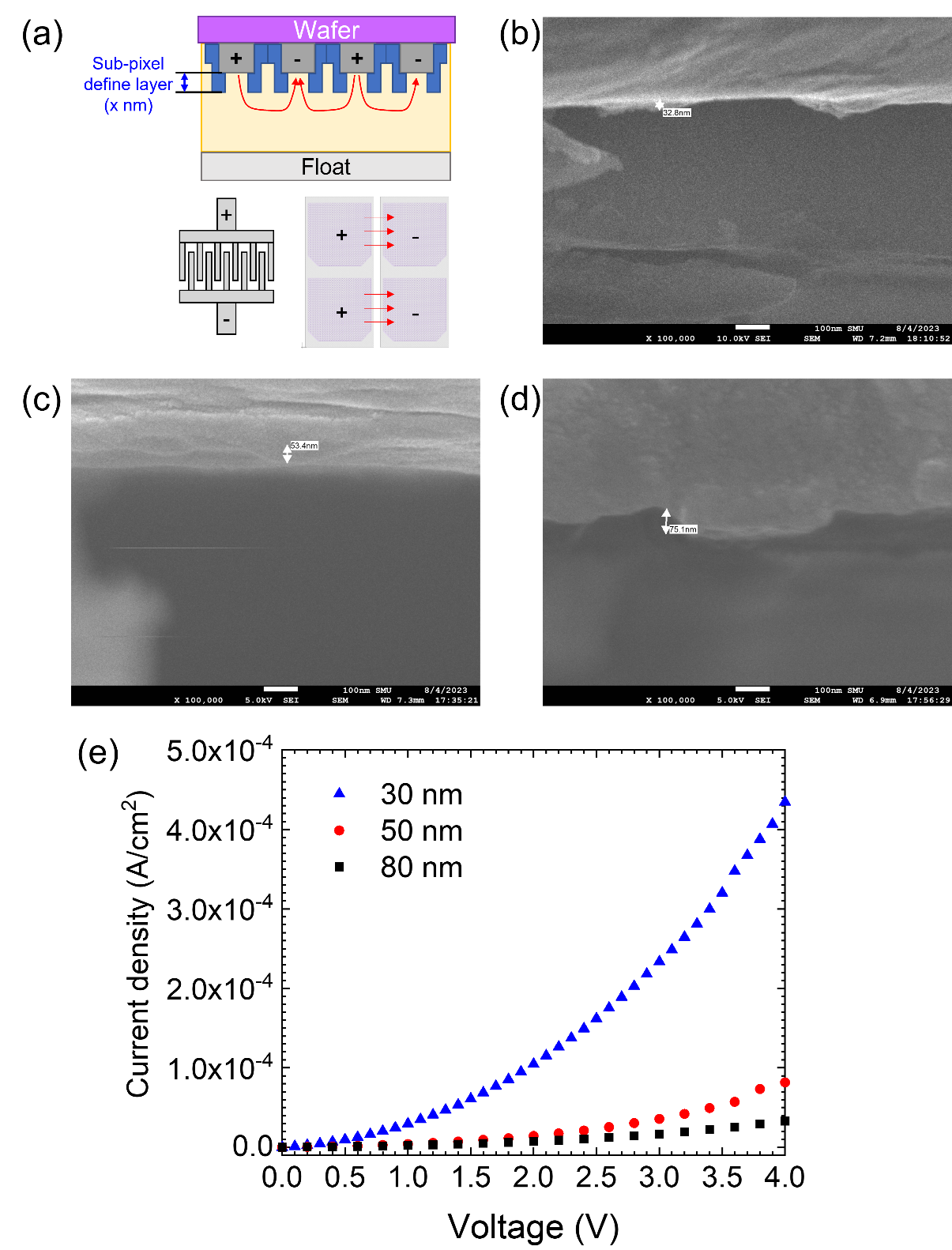


Figure S7. (a) Schematic cross-sectional structure of the circuit with finger electrodes, scanning electron microscope (SEM) images with (b) 30 nm, (c) 50 nm, (d) 80 nm thick SiO_2_ sub-pixel define layers, and (e) current-density (*J-V*) characteristics of single stack white OLED with different sub-pixel define layer thicknesses in the “Lateral” driving condition.

**References**

S1. Blakesley, J. C. et al. Towards reliable charge-mobility benchmark measurements for organic semiconductors. *Org. Electron.* **15**, 1263–1272 (2014).
